# Effect of Clearcutting Operations on the Survival Rate of a Small Mammal

**DOI:** 10.1371/journal.pone.0118883

**Published:** 2015-03-06

**Authors:** Martín A. H. Escobar, Sandra V. Uribe, Romina Chiappe, Cristián F. Estades

**Affiliations:** Laboratorio de Ecología de Vida Silvestre, Facultad de Ciencias Forestales y Conservación de la Naturaleza, Universidad de Chile, Santiago, Chile; Università degli Studi di Napoli Federico II, ITALY

## Abstract

Clearcutting is a common timber harvesting technique that represents a significant and abrupt change in habitat conditions for wildlife living in industrial forests. Most research on this type of impact has focused on comparing populations or communities in mature forests/plantations and the resulting clearcut stands. However, this approach does not separate the effect of changes in habitat attributes from direct mortality produced by the intensive use of heavy machinery required for cutting down trees and dragging them to a road. Because knowing the fate of individuals after a disturbance is important for modelling landscape-scale population dynamics in industrial forests, we conducted a study in South-Central Chile to understand the short-term response to clearcutting operations of the long-haired Akodont (*Abrothrix longipillis*), a forest specialist mouse. Between 2009 and 2013 we radiotracked a total of 51 adult male Akodonts, before, during and after the clearcutting of the pine plantations in which they lived. A minimum of 52.4% of the individuals died as a direct cause of the timbering operations, being crushed by vehicles or logs during logging operations. Our observations suggest that, instead of fleeing the area, the response of long-haired Akodonts to the approaching machinery is to hide under the forest litter or in burrows, which exposes them to a serious risk of death. The real mortality rate associated to clearcutting may be higher than that estimated by us because of some methodological biases (i.e. individuals with crushed radiotransmitters not recorded) and the fact that additional mortality sources may affect the population in the weeks following logging operations (e.g. higher exposure to predation, effects of site preparation for the new plantation, etc).

## Introduction

Clearcutting is a controversial but widespread timber harvesting technique [1,2] used in most fast-growing commercial forests, particularly conifer plantations [3,4]. Concerns regarding this technique are related to its potentially detrimental effects on soil erosion [5], nutrient cycling [6], wildlife habitat [7], alien invasions [8], and aesthetics [9], among others.

For wildlife living in these industrial forests, clearcutting represents a significant and abrupt change in habitat conditions that may render the site unsuitable for many species [10,11,12]. Several studies have shown important differences in the composition of forest wildlife after clearcutting has taken place (e.g. [13,7,14]). However, besides the obvious change in habitat attributes, such as vegetation structure and composition, microclimate, etc., clearcutting may entail a serious risk of direct mortality for many forest wildlife, because of the intensive use of machinery required for cutting down all the trees over a large tract of forest and transporting them to a road [15].

Most assessments of the effects of clearcutting on forest wildlife have been conducted at the population level, comparing attributes such as abundance, reproduction, etc., in the original forest and clearcuts (e.g. [16,17,18]). However, such approaches cannot separate the effect of the change in habitat structure and composition from the direct impact of the cutting and logging operations. In other words, most available information on the topic cannot identify the extent to what the reduction (or disappearance) of an animal population in a stand subjected to clearcutting was due to the relocation or to the death of the individuals. Knowing the fate of individuals after a disturbance is important for modelling landscape-scale population dynamics in industrial forests. Depending on how much mortality is induced by disturbance, disturbed patches may behave as temporary population sinks (i.e. most individuals die) or sources (i.e. most individuals survive but are forced to disperse) [19]. In the latter case, evicted individuals may tend to aggregate in the remaining undisturbed vegetation [20,21], potentially affecting local populations as the result of “over-compensating” density-dependence [22]. For that reason it is essential to understand the short-term response of wildlife to clearcutting operations.

Forest industry in Chile is based mostly on Monterey pine (*Pinus radiata* D. Don) plantations, which cover approximately 1.5 million hectares in the country [23]. In spite of their structural and compositional simplicity, these plantations are known to host several wildlife species [24]. This poses the problem that when these plantations are harvested there may be a large number of animals evicted from the area, and many individuals may have difficulties relocating into suitable habitat, particularly the least vagile species.

We conducted a study aimed at assessing the fate of individual long-haired Akodonts (*Abrothrix longipillis*) after the clearcutting of the pine plantations in which they live. We hypothesized that the survival of these mice would be associated to the presence of nearby potential refuges (i.e. uncut vegetation) to which they could relocate.

## Materials and Methods

### Ethics Statement

We made every attempt to reduce disturbance, stress, and other impacts to target and non-target species during the course of this research. This study was carried out in strict accordance with the recommendations of the “Guide for Environmental Evaluation of Wildlife” (G-PR-GA-03) of the Chilean and Agriculture and Livestock Service (SAG). SAG's Division of Renewable Natural Resources Protection approved this study, and all animals were captured under permits 2794 (2009), 3860 (2010), 2916 (2011) and 6513 (2013). Our study did not involve endangered or protected species.

### Study Area

**Fig 1:**
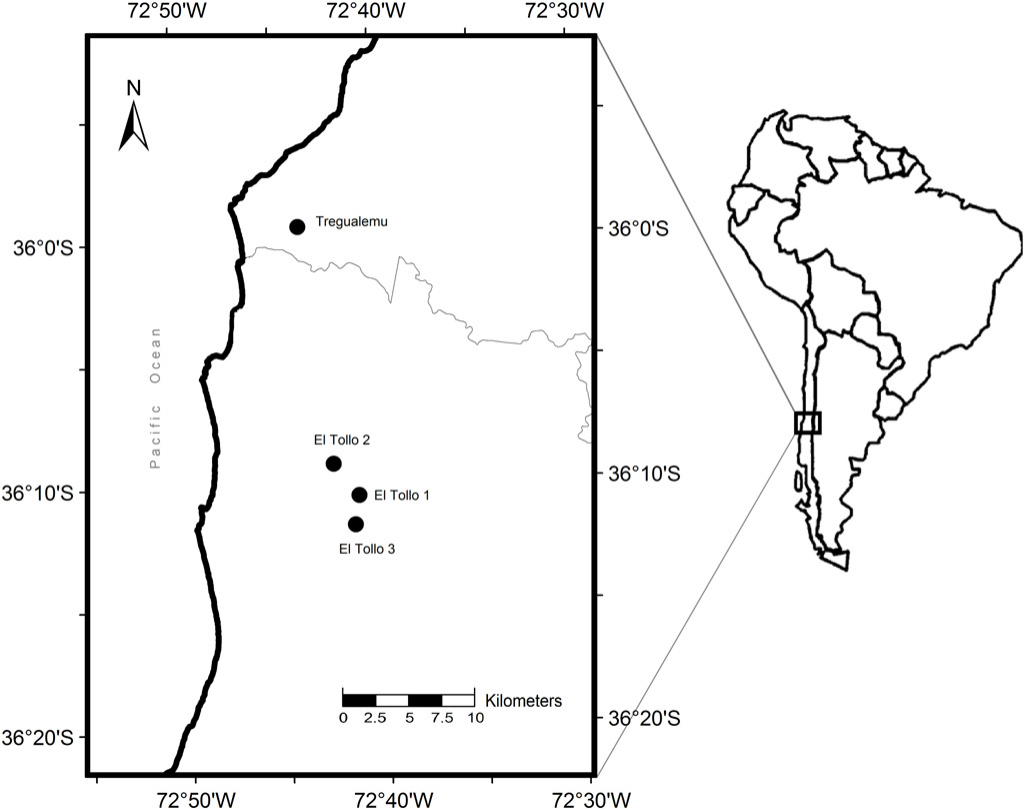
Study region showing the location of the study sites.

Our study was carried out in the coastal range of south-central Chile (Fig. 1), where climate is mild (annual average 12°C and 1,132 mm precipitation) due to the influence of the ocean [25]. The region was once covered by temperate deciduous forests dominated by *Nothofagus* spp. [26] but, during the last century it suffered a significant change due to extensive forest cutting to clear land for agriculture, followed by the substitution of scrubland and natural forests by exotic forest plantations [27]. Currently, most of the landscape is dominated by exotic Monterey pine plantations, with only a few small and scattered fragments of native forest remaining [28]. In the mentioned region we selected four pine stands that were subjected to clearcutting in the following years: 2009 (El Tollo 1, 14.2 ha) (36° 10’ 33” S; 72° 41’ 18” W), 2010 (El Tollo 2, 129 ha) (36° 09’ 42” S; 72° 42’ 28” W), 2011 (Trehualemu, 55 ha) (35° 59’ 35” S; 72° 43’ 53” W) and 2013 (El Tollo 3, 148 ha) (36° 11’ 45” S; 72° 41’ 22” W) (Fig. 1). All study sites belong to MASISA SA, who allowed us to conduct this research on their properties.

### Study species

The long-haired Akodont is an endemic rodent of Chile and Argentina [29]. In Chile, it is distributed from 28° to 56° latitude South [30]. Although it is described as a typical forest species, it also thrives in exotic pine plantations [31], and our observations in the study area indicate that this mouse is the most abundant small mammal in mature plantations. The long-haired Akodont has a high site fidelity [32,33] which might render this species as especially sensitive to disturbances such as clearcutting.

### Capture and radiotelemetry

All the field work was conducted during the austral winters (June-August) as most clearcutting operations in this region take place during the wet season. Two to three weeks before the initiation of the harvest, we captured long-haired Akodonts with live traps (3 x 3.5 x 9", aluminum and mesh) baited with rolled oats [34]. We set traps during three nights placing the traps in different locations within the stand in order to obtain individuals at different distances from the refuges (uncut vegetation).

For each captured individual we recorded its body mass, sex and age class. In order to reduce the variability of the sample, only adult males (>36 g) were selected for this experiment. These individuals were all ear-tagged, equipped with a 3.6 g radio-transmitter with mortality sensor (TXD-110C, Telenax, Playa del Carmen, México), and released in the same point of capture. The location of the capture site of each individual was recorded with a GPS unit (5 m accuracy) and it was used to estimate the distance to the nearest refuge (uncut vegetation).

We used a portable receiver (R-1000, Communication Specialists, Inc., California, USA) equipped with handheld 3-element Yagi antennas (RA-150, Communication Specialists, Inc., California, USA) to locate radio-collared individuals. Animal locations were estimated from triangulations using two or more bearings obtained simultaneously from fixed telemetry stations (out of 28–32 stations) every night [35], because the species is mostly crepuscular and nocturnal [30], and because we had to get into the sites once the loggers' shift had ended. We monitored the condition (alive or dead) and movements of the individuals from 2–3 weeks before harvest to up to a week after the operations had finished. Once the experiment had ended, we made an effort to recapture the studied individuals in order to rid them from the radio collars. We estimated the location of each individual by maximum likelihood estimation in LOAS (4.0.3.8) using error polygon size of 0.1 ha. Then, we estimated home range by fixed kernel (75%) [36] in program BIOTAS (2.0a) (Ecological Software Solutions). During the first two years of study we were able to estimate the individuals' location on a daily basis. However, for the last two seasons safety regulations prevented us from working every night so we did not include these data in the home range analysis.

### Survival analysis

When a transmitter emitted a mortality pulse, we searched and recovered it, in order to determine the cause of death or if the transmitter had detached from the individual. We classified deaths into the following classes: natural cause (i.e. no visible signs of an external agent), predation (i.e. evident signs of having been attacked by another animal), or crushing (i.e. crushed body or other signs of mechanically induced injuries). Individuals whose signals were lost were classified as missing and were not considered for the analyses. With a *G* ^*2*^ test [37] we tested if there were significant differences in the proportion of individuals surviving or dying from different causes between years.

The mortality sensor in the transmitters allowed us to estimate the daily survival of individual mice. We compared the daily survival probability for three stages during the harvesting season: pre-logging (no presence of machinery or personnel in the individual's home range), logging (tree felling and logging taking place within the home range) and post-logging (no major operations in the home range). The first stage was 15 (± 5) days long, whereas the logging phase lasted 3 days. Finally, we monitored individuals for 6 days after logging had ended. We estimated the mean daily survival rates using the modified Trent and Rongstad estimator [38,39]. Differences between survival rates were tested using confidence intervals at the 95% confidence level, estimated by a method for determining the differences between two extremely small binomial probabilities [40].

Finally, we used a logistic regression [37] to test for an effect of the proximity to potential refuges on individual survival after clearcutting. We performed all statistical analyses with INFOSTAT [41].

## Results

We captured and radio-tracked a total of 67 adult male long-haired Akodonts during the four years of study. Average (± SD) individual weight was 51.5 ± 9.1 g, and the average (± SD) proportion of the transmitter weight respect to body weight was 7.2 ± 1.2% (all cases < 10%). We radio-tracked the individuals 11.2 ± 3.3 days (mean ± SD) with a range to 8–21 days before harvest to home range analysis, and the size of individuals' home range was 0.8 ± 0.2 ha (mean ± SD).

**Fig 2:**
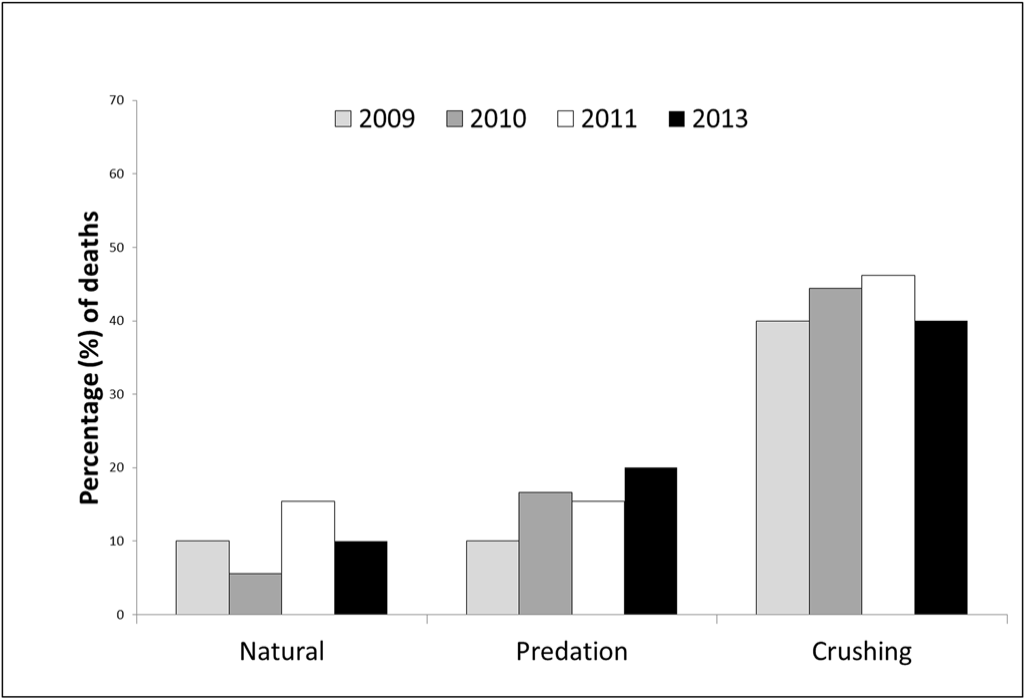
Percentage of deaths by different causes for adult males of long-haired Akodont, during the four years of study.

The signal of 16 transmitters was lost during the experiment (6, 6, 2 and 2, for the years 2009, 2010, 2011 and 2013, respectively) and the corresponding individuals were, consequently, excluded from the analyses. Table 1 shows the condition of all individuals at the end of the experiment for each monitored stage. During the Pre-logging stage, 17.6% (9/51) of the analyzed long-haired Akodonts died of natural (unknown) causes (11.7%) or due to predation (5.9%). During the logging stage, 52.4% (22/42) of the remaining individuals died, apparently, as a direct result of the logging operations in the plantations in which they lived. Finally, during the post-logging stage, 20.0% (4/20) of the remaining individuals died of natural causes (5%) or due to predation (15%). Over four years of study, the proportion of different mortality causes were very similar among years (Fig. 2), and *G* ^*2*^ test showed no interannual differences among the latter frequencies (*G* ^*2*^ = 1.74; p = 0.99).

**Table 1:** Fate of adult male long-haired Akodonts after the clearcutting of the pine plantations in which they lived.

Stage	N	Alive		Dead					
				Natural		Predation		Crushing	
		n	%	n	%	n	%	n	%
**Pre-logging**	51	42	82.4	6	11.7	3	5.9	-	-
**Logging**	42	20	47.6	-	-	-	-	22	52.4
**Post-logging**	20	16	80.0	1	5.0	3	15.0	-	-

Total individuals at the beginning of each stage (*N*), number of subjects (*n*) and percentages (*%*) in each category.

**Fig 3:**
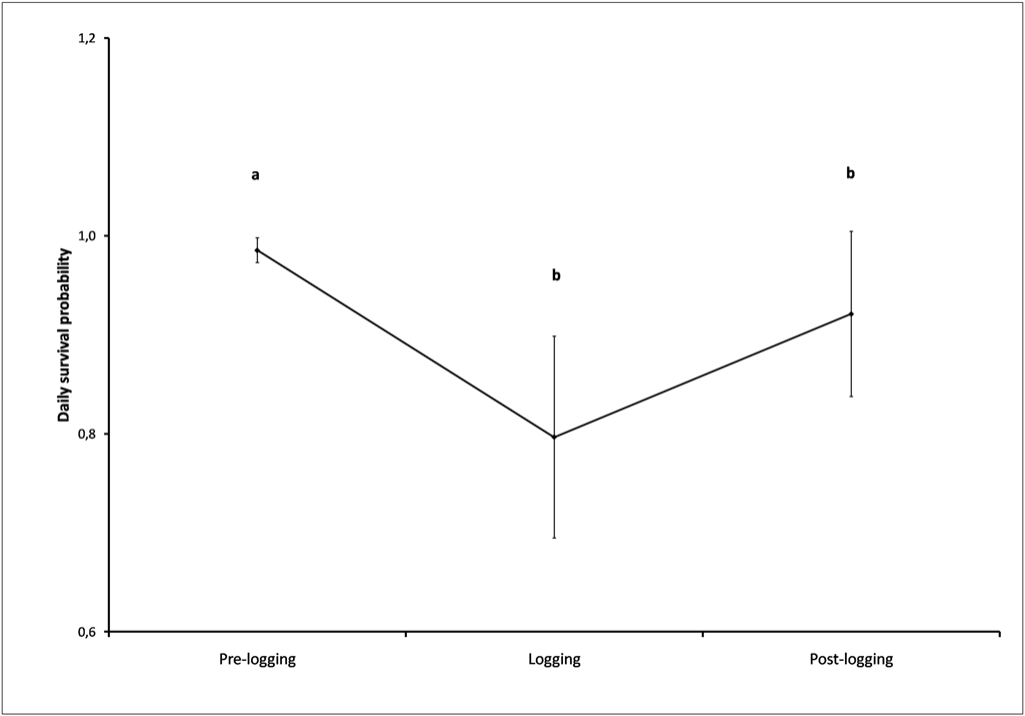
Daily survival probabilities for adult male long-haired Akodonts, before, during and after the logging of the pine plantations in which they lived (2009–2010). Bars indicate standard error. Different letters on the top of each value indicate significant differences of contrast tests at a 95% confidence level.

Most of the recovered dead mice had, apparently, been hiding underneath the forest litter or in burrows when they were crushed by the skidders or timber harvesters. In one case we had to dig up to 50 cm in the ground to recover a dead Akodont whose burrow had collapsed under the weight of the machinery. In other cases, the individuals seem to have died crushed by the logs that were dragged around the site. In fact, for six individuals we could only assume the cause of death because we could not reach to their bodies, as the signals came from underneath big piles of logs or harvest debris. The comparison of the Trent and Rongstad estimators [38] for the three experimental stages (Fig. 3), showed that there was a significant reduction in the Akodont survival rate from the pre-logging to the logging phase (Z = 6.24, p < 0.05, n = 28). Survival rate increased slightly after logging operations had ended, but it was still significantly lower than during the pre-logging phase (Z = 2.44, p < 0.05, n = 28).

The average (± SD) distance from the capture site of each individual to the nearest refuge (uncut vegetation) was 127.2 ± 79.2 m, and the range distance was 18–290 m. The logistic regression between the survival of individuals and the distance from their capture site to the uncut vegetation showed that the survival was higher closer to refuges, but this trend was only marginally significant (p = 0.095).

## Discussion

Our study is among the very few that document the short-term effects of logging operations on vertebrates. Reinert et al. [15] studied the response of timber rattlesnakes to mechanized lumbering operations in Pennsylvania, finding a rather negligible mortality (<2%) associated with the process. Interestingly, most of the reported deaths were due to the deliberate killing of snakes by workers. In Quebec, Ferron et al. [42] radiotracked snowshoe hares in an area subjected to clearcutting with protection for regeneration. These authors did not find any significant differences between the mortality rate before and after harvesting, and all of the documented deaths were due to predation. Also in Canada, Turcotte et al. [43] followed spruce grouses before, during and after forest cutting, finding that mortality was actually reduced after logging operations, with predation being its main cause.

In contrast to the latter, our results show that the long-haired Akodonts experience an important mortality rate during plantation harvesting operations, with more than 50% of the adult males individuals dying of injuries caused by the movement of machinery and logs. Moreover, ours could be a conservative estimate as some of the missing individuals could be the result of our failure to detect the signals from mice buried deep into the soil, or because transmitters were also destroyed during the experiment.

High off-road vehicle activity has been reported to reduce survival of deer mice in Utah [44]. Although the movement of heavy machinery during timber logging has been shown to affect the habitat of several soil invertebrates through soil compaction [45,46], estimating the mortality of fossorial vertebrates due to burrow collapse is a more difficult task [47]. Mendonça et al. [48] intentionally collapsed gopher tortoise burrows with a skidder and showed that all individuals survived, even if trapped for several days, and that most were able to self excavate.

The behavior of long-haired Akodonts seems to conspire against their survival during timbering operations. We had hypothesized that the closeness of potential refuge (uncut vegetation) would increase the chances of individuals to survive after clearcutting. However, we did not find such an effect, likely because our implicit premise that individuals would try to leave the area when facing danger, appears to be wrong. Rather than fleeing the area, the prevalent response of long-haired Akodonts is to hide underneath slash, forest litter or in burrows, exposing them to being crushed by machinery.

The presence of refuges may be important for the survival of animals living in environments subject to intense disturbances [49]. Thus, small refuges such as dense shrubby spots, rock outcrops, etc., within the clearcut area, could favor the survival of the less vagile animals. Although we did not conduct a formal assessment on the latter, we observed that some of the surviving Akodonts were associated to spots where the microtopography had apparently prevented skidders from running over them.

Although there must be an obvious species effect, part of the explanation for the important differences in mortality rates observed in our study compared to the northern ones [15,42,43] may be the fact that, in the latter, a proportion of the trees was left standing, whereas in our case a complete clearcutting was conducted (i.e. all trees were removed). These contrasting results support the potential favorable effect of refuges on individual survival. For instance, Reinert et al. [15] observed that over 30% of timber rattlesnakes retreated from the logging activity and sheltered under remaining ground cover.

However, there might be a potentially negative, and counterintuitive, effect of refuges on animal survival during a disturbance. Normally the presence of nearby vegetation cover, burrows, etc., creates a sensation of safety in animals [50], which may decide not to escape from danger but to hide. Although this behavior may provide a selective advantage as a predator avoidance strategy, it may also make individuals more vulnerable to infrequent but destructive events such as clearcutting. Most long-haired Akodonts are likely used to having several refuges accessible within their home ranges, since most pine plantations in our study region have a very complex understory [24]. This would likely exacerbate their tendency to not run from danger.

Although the impact of heavy logging machinery on variables such as soil compaction and erosion, water quality, etc., has been thoroughly analyzed [45,46,51–53], the role of the use of such equipment on wildlife mortality has been, apparently, overlooked. For example, Semlitsch et al. [54] proposed different mechanisms by which timber harvesting might affect forest amphibians, none of which includes the direct impact of logging operations on the mortality of individuals.

We only followed long-haired Akodonts for a few days after logging had ended. That means that additional mortality can occur during the following weeks. Besides the higher exposure to predators in open areas [55,56], the effects of slash management and other site preparation activities (ploughing, use of herbicides, etc.) may exert an important mortality on the remaining individuals [57,58].

Including our study, the available evidence suggests that the short-term effects of logging on wildlife are likely species-specific and strongly dependent on the silvicultural treatments being used. That implies that a much larger body of information on this topic is needed in order to correctly assess the effects of forest management on wildlife both at the local and landscape scales.
